# Time-resolved nuclear dynamics in bound and dissociating acetylene

**DOI:** 10.1063/1.5037686

**Published:** 2018-08-20

**Authors:** C. Burger, A. Atia-Tul-Noor, T. Schnappinger, H. Xu, P. Rosenberger, N. Haram, S. Beaulieu, F. Légaré, A. S. Alnaser, R. Moshammer, R. T. Sang, B. Bergues, M. S. Schuurman, R. de Vivie-Riedle, I. V. Litvinyuk, M. F. Kling

**Affiliations:** 1 Department of Physics, Ludwig-Maximilians-Universität München, D-85748 Garching, Germany; 2 Max Planck Institute of Quantum Optics, D-85748 Garching, Germany; 3 Centre for Quantum Dynamics and Australian Attosecond Science Facility, Griffith University, Nathan, QLD 4111, Australia; 4 Department of Chemistry and Biochemistry, Ludwig-Maximilians-Universität München, D-81377 Munich, Germany; 5 Énergie, Matériaux et Télécommunications, Institut National de la Recherche Scientifique, Varennes, Quebec J3X 1P7, Canada; 6 Université de Bordeaux-CNRS-CEA, CELIA, UMR 5107, F-33405 Talence, France; 7 Department of Physics, American University of Sharjah, PO Box 26666, Sharjah, United Arab Emirates; 8 Materials Science and Engineering Research Institute, American University of Sharjah, PO Box 26666, Sharjah, United Arab Emirates; 9 Max Planck Institute of Nuclear Physics, D-69117 Heidelberg, Germany; 10 National Research Council of Canada, 100 Sussex Dr, Ottawa, Ontario K1A0R6, Canada

## Abstract

We have investigated nuclear dynamics in bound and dissociating acetylene molecular ions in a time-resolved reaction microscopy experiment with a pair of few-cycle pulses. Vibrating bound acetylene cations or dissociating dications are produced by the first pulse. The second pulse probes the nuclear dynamics by ionization to higher charge states and Coulomb explosion of the molecule. For the bound cations, we observed vibrations in acetylene (HCCH) and its isomer vinylidene (CCHH) along the CC-bond with a periodicity of around 26 fs. For dissociating dication molecules, a clear indication of enhanced ionization is found to occur along the CH- and CC-bonds after 10 fs to 40 fs. The time-dependent ionization processes are simulated using semi-classical on-the-fly dynamics revealing the underling mechanisms.

## I. INTRODUCTION

The ionization of molecules with intense laser pulses typically results in the vibrational and electronic excitation of the molecule. Upon ionization, the molecular structure and the corresponding chemical properties can change drastically, e.g., due to proton migration,^1^ dissociation,^2^ or even selective bond-breaking.^3^ To temporally resolve the resultant vibrational motion, Coulomb explosion imaging (CEI) can be applied to reveal the underlying nuclear dynamics.

**FIG. 1.**
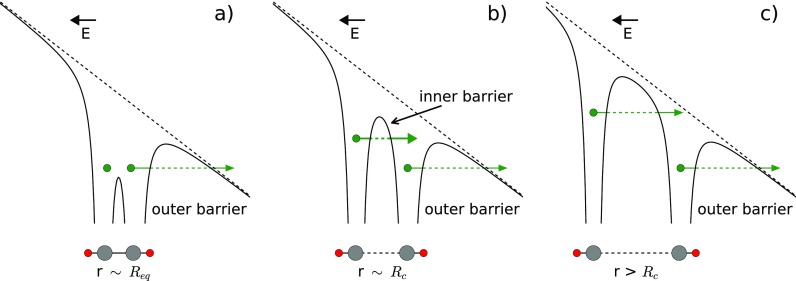
Schematic representation of enhanced ionization: Molecular potentials (black solid lines), which are dressed by the laser field (E, black dashed lines), for different interatomic separations: (a) at the equilibrium distance *R*_eq_, (b) at a critical distance *R*_c_, and (c) at even larger separations *R* > *R*_c_. Electrons are depicted as green points with arrows indicating tunnel ionization through the barriers. Below each panel, as an example, enhanced ionization with respect to the CC-bond in acetylene is depicted with carbon (grey) and hydrogen (red) atoms.

The ionization process itself can also be investigated by the CEI method. The enhanced ionization (EI) process is known to alter the ionization probability of molecules at an inter-nuclear distance of around twice the equilibrium bond length.^4,5^ The process of double ionization via EI can be understood for the diatomic case as follows (see Fig. 1):^4^ First, a diatomic potential well, with an inter-nuclear distance of *r* being the equilibrium distance *R*_eq_, is tilted by a strong laser field such that an electron can tunnel through the outer barrier into the continuum, cf., Fig. 1(a). Upon nuclear motion induced by the first ionization, the inter-nuclear distance increases, while an inner barrier between both nuclei emerges. At a critical distance *R*_c_, the (inner) barrier towards electron tunneling becomes smaller than the outer barrier, considerably enhancing the rate for double ionization (enhanced ionization), cf., Fig. 1(b). When the inter-nuclear distance increases further, the inner barrier becomes larger and enhanced ionization ceases, cf., Fig. 1(c). This simple picture, while illustrative, may be incomplete for the case of polyatomic molecules where multiple bonds are present. In the case of acetylene, both the CC-bond (depicted in Fig. 1) and the CH-bonds can elongate as a result of the first ionization step, providing a multi-dimensional landscape for enhanced ionization.

In 1995, Zuo and Bandrauk^6^ theoretically predicted enhanced ionization in H_2_^+^ ions for a charge resonance between two strongly coupled states. As this process depends on the inter-nuclear distance, which may change following the first laser-molecule interaction, its temporal evolution is of particular interest. Ergler *et al.* performed first time-resolved pump-probe measurements on hydrogen,^7^ followed by more extensive work on H_2_ (Refs. 8 and 9) and on D_2_.^10^ Another method of probing EI is to change the pulse duration^11–13^ or the polarization of the laser^14^ and thereby investigate the role of EI on the ionization probability. Wu *et al.* verified that in the process of EI the potentially high-lying nucleus is ionized using circularly polarized pulses.^4^ Theoretical calculations were able to reproduce EI both in simple hydrogen^15^ and in more complex acetylene^16,17^ showing the importance of two or more interacting states where charge-resonance enhanced ionization (CREI) can occur.

Experimental investigations on enhanced ionization of polyatomic molecules are still scarce. Roither *et al.* found that various hydrocarbons can efficiently be ionized (up to charge states of +12) via enhanced ionization.^18^ This enhancement process is mediated by the stretching motions of the CH-bonds and occurs at bond lengths, which are about twice as large as the equilibrium value.^18,19^ Recently, Erattupuzha *et al.* found that CREI in acetylene originates not only from coupling of two states but rather due to an energy upshift and field coupling of multiple orbitals.^20^ Following this argument, EI in complex molecules such as acetylene is not limited to a single critical inter-nuclear distance but can include a broader range of inter-nuclear distances, which are possibly assumed during a longer time interval during molecular motion initiated by, e.g., the ionization of the neutral molecule.

In previous pump probe experiments, light-induced vibrational motion in neutral systems and molecular ions was imaged by time-dependent ionization spectroscopy. These experiments were predominantly performed for rather simple diatomic systems (see, e.g., Refs. 21–23), but also for more complex molecules including small hydrocarbons.^24–27^

In this work, strong-field ionization induced by the pump-pulse prepares a wave packet in cationic or dicationic states of acetylene. The motion of this wave packet is detected by a second ionization via the probe pulse. Our study allows us to investigate both enhanced ionization as well as vibrational motion in charged states of acetylene within the same time-resolved experiments. The joint experimental and theoretical work provides insight into the nuclear dynamics in bound and dissociating molecules.

## II. METHODS

### A. Experimental methods

In our time-resolved experiments, a femtosecond pump-probe setup was utilized, as described in Ref. 28. In brief, a few-cycle laser pulse with <5 fs pulse duration was split into two pulses in a Mach-Zehnder interferometer. The temporal delay between both pulses was adjusted by a linear nm-resolution translation stage in one arm of the interferometer. The delay was continuously swept between 0 fs and 120 fs, with one complete scan taking around 5 min, whereas the total scan duration was about 12 h with average count rates around 5 kHz. By analyzing the individual short-term scans, long-term drifts in the delay could be identified and compensated. The pulse duration of the individual arms was measured independently by a frequency-resolved optical-gating technique (FROG) behind the interferometer.^29^ Additional laser parameters such as spectrum, power, and focal spot size were recorded before and after the scan to ensure similar conditions throughout the measurement. Both laser pulses were subsequently intersected with neutral acetylene molecules in the jet of a reaction microscope (REMI). The REMI provides the 3D momentum distributions of all charged particles resulting from the laser-molecule interaction. In our experiments, we focused on measurements of emitted fragment ions, with a momentum resolution of the order of 0.1 a.u. for the present experimental conditions. The detection of coincident fragment ions from the dissociation of the molecule induced by both pulses is used to separate fragmentation channels. Each few-cycle pulse had an intensity of around 5 × 10^14^ W cm^−2^, permitting single or double ionization of acetylene by a single pulse. The pulse intensities were calculated from the pulse energies, pulse durations, and focus size. They have an uncertainty of about 20%.

### B. Computational details

The time-dependent ionization processes were calculated using a combination of non-adiabatic on-the-fly simulations and *ab initio* calculations of ionization rates. For all this, the Complete Active Space Self-Consistent Field method (CASSCF)^30^ was employed. The active space provided for the static electron correlation was adapted to the number of the valence electrons consisting of 10 molecular orbitals and the according electrons except for the 1s-core electrons of the carbon atoms, following Refs. 31 and 32. The calculations were performed using the program package MOLPRO 2012 (Refs. 33 and 34) with the 6–311++G** basis set.^35–37^ For the non-adiabatic on-the-fly simulations, we used a modified version of Newton-X,^38–40^ which supports the usage of MOLPRO 2012.

Sets of, respectively, 100 trajectories were propagated in the ground state of the acetylene cation (X^2^Π_u_) and the first dissociative state of the dication (A^3^Π_u_), both starting from a Wigner distribution around the ground-state equilibrium geometry. The propagation time for the A^3^Π_u_ state was 100 fs and for the X^2^Π_u_ state was 200 fs, both with a step size of 0.25 fs. For 20 typical trajectories, the ionization probability (hereafter called rate) was calculated with the ansatz described in Refs. 41 and 42. In this ansatz, a quantum chemical calculation for a given molecular geometry with and without a static electric field is performed. Based on the obtained two electronic densities, the tunneling rate can be extracted. For acetylene, the highest three occupied orbitals are included to build up the electronic densities. In the current work, an angular dependence for the ionization was not included. The methodology is benchmarked against the more rigorous time-dependent resolution in ionic states (TD-RIS) approach,^43–45^ see Appendix A. Additionally, 60 trajectories with 200 fs propagation time were calculated in the A^2^Σ_g_^+^ state of the cation, see Appendix B.

## III. RESULTS AND DISCUSSION

**FIG. 2.**
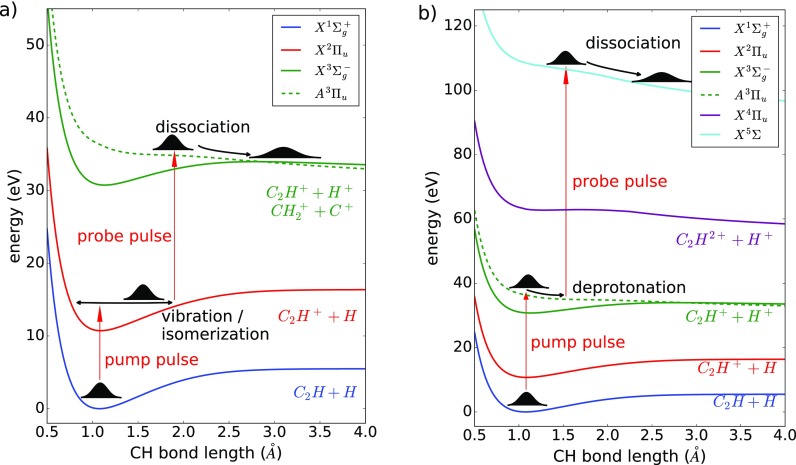
Potential energy curves for various states in neutral and various charged states of acetylene as a function of CH-bond length. In scenario (a), the pump pulse ionizes the molecule into a bound cation state and a subsequent probe pulse populates a dissociative dication state (here the first excited state of the dication). In scenario (b), the pump pulse directly populates the dissociative dication state, and the probe pulse induces fragmentation from a quadruply charged molecular state.

In our analysis, we focus on two scenarios for the pump-probe experiment that are displayed in Fig. 2. In the first scenario, the pump pulse populates the bound ground state of the acetylene cation, where the wavepacket starts to oscillate, see Fig. 2(a). The wavepacket dynamics is probed by further dissociative ionization with the probe pulse. Here, the deprotonation (C_2_H_2_^2+^ → H^+^ + C_2_H^+^) and isomerization (C_2_H_2_^2+^ → C^+^ + CH_2_^+^) are two product channels that are observed after dissociative double ionization. In the second scenario, the pump pulse directly populates a dissociative dication state [e.g., the first excited state as displayed in Fig. 2(b)]. Previous work indicated that at similar laser intensities, this process was facilitated by recollisional ionization/excitation after single ionization.^47^ The probe pulse enables further ionization, including [as shown in Fig. 2(b)] the generation of the dissociative quadruply charged molecule, which fragments by Coulomb explosion. In our studies, we focus on the observation of fragments from the quadruply charged instead of a triple charged molecule, since it enables a better probe for the dissociative dynamics on the dication excited state due to its steeper potential and hence stronger energy difference as a function of bond length.

### A. Nuclear dynamics in bound electronic states of the cation

To investigate the temporal evolution of the nuclear motion in the bound states of the cation created by the pump pulse, we analyzed the kinetic energy release (KER) of the ionic fragments created by the probe pulse as a function of the delay between both laser pulses.

**FIG. 3.**
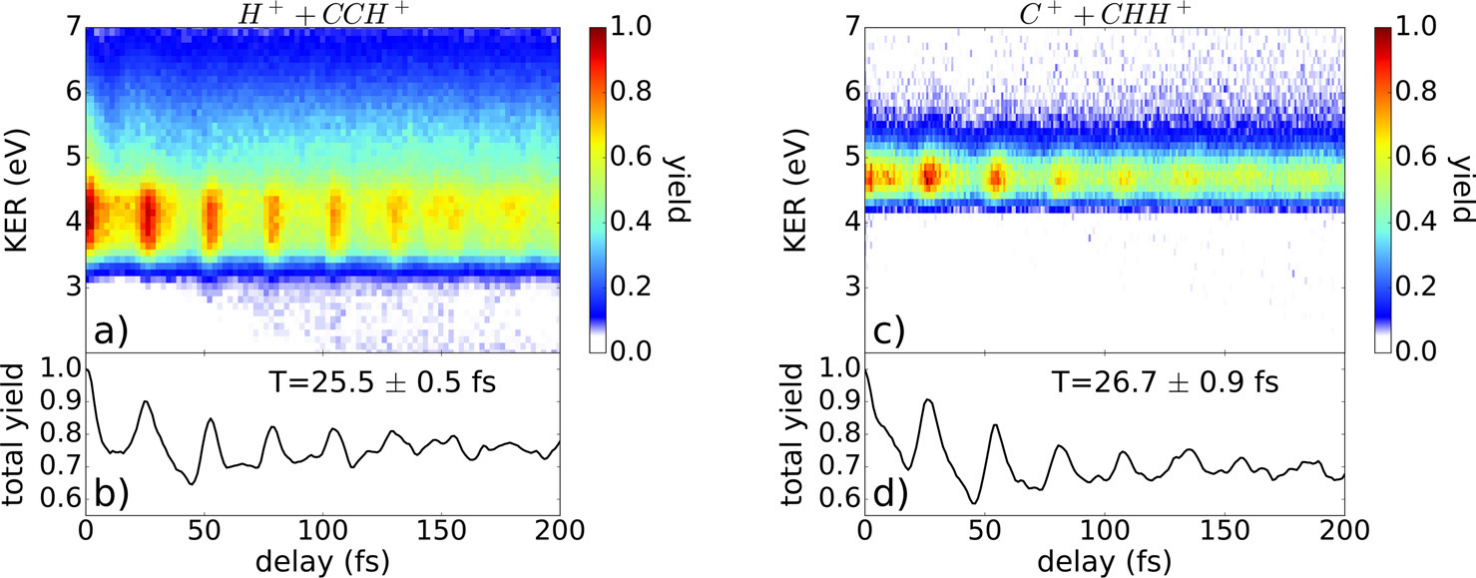
Ionization yield of (a) the deprotonation and (c) isomerization channel as a function of the kinetic energy release (KER) and the delay between pump and probe pulse. (b) and (d) The KER-integrated signal as a function of delay.

In Fig. 3, the ionization yield is depicted as a function of KER and delay for the deprotonation channel [Fig. 3(a)] and the isomerization channel [Fig. 3(c)]. Since the probe step populates a dissociative dication potential [cf., Fig. 2(a)], the KER is an indicator of the bond length in the cation, where a short bond length results in a higher KER and vice versa. As visible in Figs. 3(a) and 3(c), the KER remains rather constant over time, which is a strong indication of nuclear dynamics in bound electronic states of the cation. As already discussed in Refs. 48 and 49, the observed KER signals suggest that the deprotonation and the isomerization processes take place in dicationic states of acetylene.

The overall temporal evolution of the ionization yield is similar within both investigated channels and a considerable modulation is observed, compare Figs. 3(b) and 3(d). The oscillations reflect the dynamics in the intermediately populated bound electronic states of the cation. This is supported by the fact that the observed vibrational periods are the same within the experimental error. An oscillation period of 25.5 ± 0.5 fs is found in the deprotonation channel and of 26.7 ± 0.9 fs in the isomerization channel. The dynamics in the intermediate states of the cation is convoluted with the transition probability to the dication states in the probe step. The TD-RIS calculations allow for the resolution of single state contributions to the total ionization yield. These results show that the states populated with the largest amplitude (from ionization of acetylenic structures) are the lowest lying bound states (X^3^Σ_g_^−^ and ^1^Δ_g_) in the dication, while higher lying states that lead to either CC or CH-bond breaking contribute very little to the total yield (see Appendix A). The subsequent isomerization and/or fragmentation leading to the experimentally observed products might be induced by coupling to dissociative states, like the A^3^Π_u_ state. The coupling could be realized by populating highly excited vibronic bound states directly during the ionization or by mixing these states due to interaction with the remaining probe laser field.

**FIG. 4.**
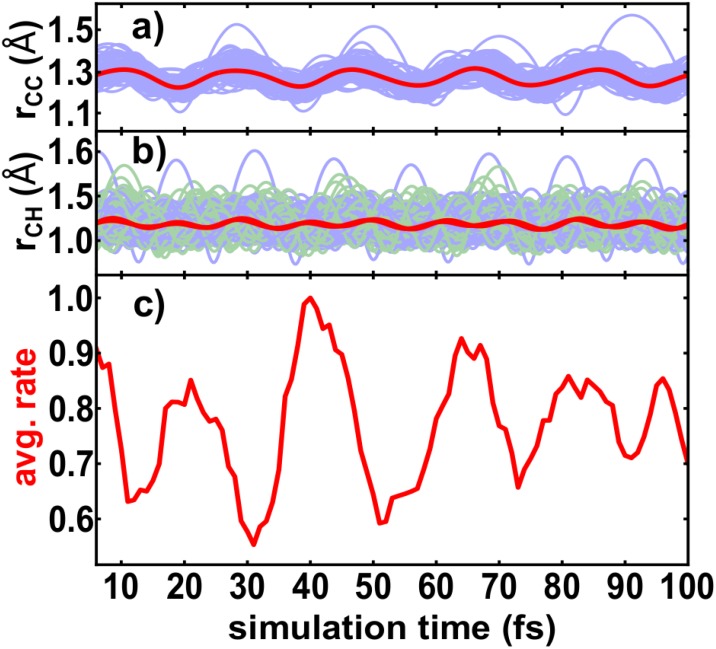
(a) Simulated temporal dynamics of the CC bond in the ground state of the acetylene cation shown in blue for all trajectories and in red for the averaged value. (b) The temporal evolution of both CH bonds in the ground state of the acetylene cation displayed in blue and green for the left and right CH bond, respectively, for all trajectories, and in red for the averaged values. (c) Calculated time-dependent averaged ionization rate of 20 exemplary trajectories propagating in the ground state of the acetylene cation.

To explain the observed periods in the ionization yields, the nuclear dynamics in the ground state of the acetylene cation (X^2^Π_u_) were simulated. The theoretical results for the dynamics of the acetylene cation are summarized in Fig. 4.

All bonds in the acetylene cation show clear oscillation dynamics, see Figs. 4(a) and 4(b). The CC bond length varies between 1.1 Å and 1.5 Å (equilibrium distance around 1.2 Å), while the length of both CH bonds varies between 0.9 Å and 1.6 Å (equilibrium 1.06 Å). The averaged CC-bond has a vibrational period of about 20 fs, while both CH-bonds oscillate with 15 fs. The averaged CH bonds are moving nearly in phase, observable by the almost perfect overlap of both red solid lines in Fig. 4(b).

The average ionization rate was calculated for 20 exemplary trajectories, see Fig. 4(c). A clear periodic modulation of the ionization rate was observed with a period of around 23 fs. The combined motion of the CC- and both CH-bonds induces the observed periodic change of the ionization rate. Comparing the averaged values of r_CH_ and r_CC_ around the peaks of the ionization rate, an anticyclic behavior can be seen: Either the CH bonds are or the CC bond is elongated, while the other bond-type is shortened. In total, the modulation of the ionization rate is dominantly determined by the symmetric CC-stretching normal mode involving motions of all four atoms.

Compared to the experimental results [see Figs. 3(b) and 3(d)], the simulated ionization rate for the ground state of the cation is in qualitatively good agreement. The vibrational period of around 23 fs is slightly shorter than the experimental one with 25 fs. A reason might be that the simulation for the ionization process focuses on the non-reactive ground state, the X^2^Π_u_ state, in which the π-bond is weakened. This is a simplification as likely not only the ground state is populated but rather also a multitude of states in the cation. We neglect the situation for which the ionization weakens the σ-bond, for example, the A^2^Σ_g_^+^ state. To estimate its possible influence, dynamic simulations for the excited A^2^Σ_g_^+^ state were performed. In this state, the oscillation period of the CC stretching motion changes due to the isomerization process. Its period varies between 22 and 25 fs within the first 100 fs (see temporal evolution of the CC bond in Appendix B). Contribution from this state might explain the slightly longer periods in the experiment. In addition, also the underlying CASSCF methodology overestimates slightly the steepness of the potential energy curve leading to a faster oscillation.

The simulations show that the vibrational motion in the bound ground state of the cation leads to periodic enhancement of subsequent ionization, which experimentally manifests in the periodic yield modulation. These results are in accordance with the experiments performed on hydrogen^50^ and deuterium.^22,51^

### B. Enhanced ionization in dissociating electronic states of the dication

Enhanced ionization for dissociating molecules is observed in the second scenario, where initially a dissociative state of the dication is populated by the pump pulse. The temporal evolution of this wavepacket is then followed by further ionization through the probe pulse. Here, we inspected the four-fold coincidence channel C_2_H_2_^4+^ → H^+^ + C^+^ + C^+^ + H^+^ that results from interaction with both pulses.

**FIG. 5.**
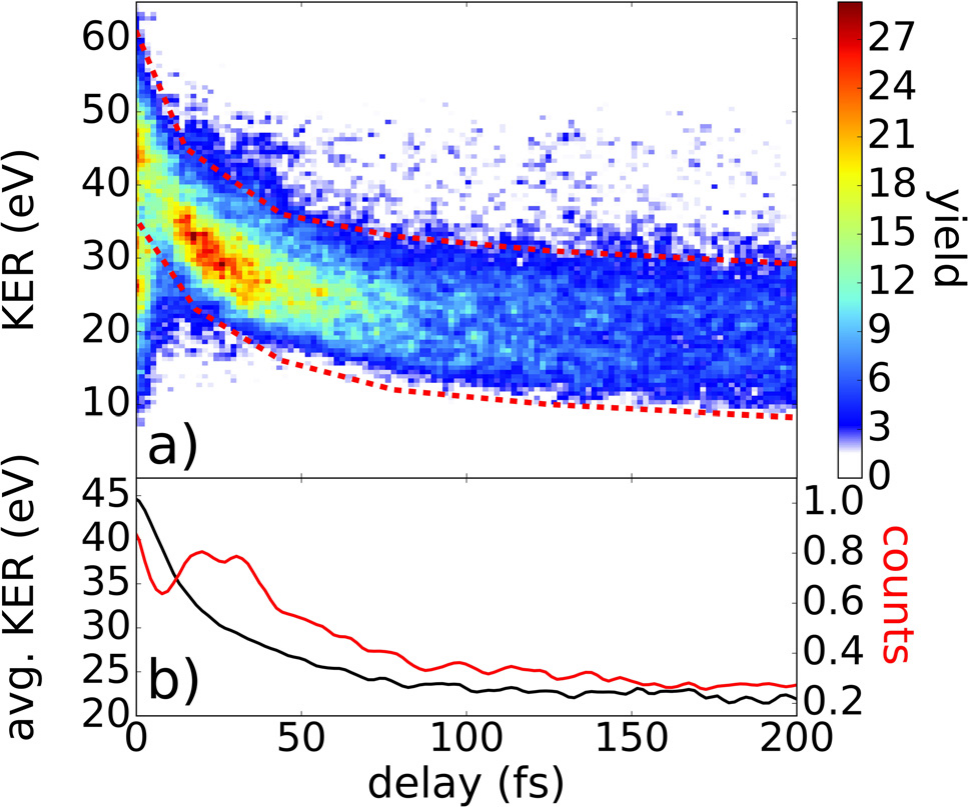
(a) Ionization yield of the four-fold coincidence as a function of KER and delay. (b) The one-dimensional plot represents the normalized KER-integrated signal including all counts within the dissociative area (red line) and the average KER of the dissociative events as a function of the delay (black line). To select dissociative molecules, only events within the red dashed lines are considered.

In Fig. 5(a), the ionization yield of the four-fold coincidences from this channel is shown as a function of KER and delay between pump and probe pulses. The area with red dashed lines corresponds to measured signal from dissociating molecules. The one-dimensional representation depicted in Fig. 5(b) shows the integrated signal (red) and average KER (black) for dissociating molecules.

The average KER is decreasing with the delay time from about 45 eV to 22 eV. Compared to Fig. 3, almost no delay independent KER contribution was detected, which indicates that all molecules detected in this channel undergo dissociation in the intermediate state, and furthermore that the pulse intensities are sufficiently low to suppress background from a single pulse.

Regarding the temporal evolution, we can also conclude that the applied pulses are short enough to separate the maximal yields at the temporal overlap and the subsequent peak starting at 10 fs. Previous time-resolved measurements on EI suffered from interferences of 2.7 fs period created by pre- and post-pulses.^7–10^ Here, however, the experimental data are not influenced by any interference due to a very clean temporal profile. As the maximum yield at the temporal overlap is not due to an enhanced ionization effect but rather stems from the increased intensity of both pulses together, this effect is not captured by the simulations.

With respect to the ionization yield in Fig. 5(a), we could observe a clear indication of enhanced ionization in acetylene. Comparing to the previous measurements performed on H_2_ (Refs. 7–9) and D_2_,^10^ the temporal evolution of acetylene shows very similar behavior, i.e., a maximum in the ionization yield within the signal of the dissociating molecules, indicating enhanced ionization of dissociating molecules. Around the temporal overlap, a strong signal is detected, which decreases with time. After a few fs, the ionization yield increases from about 10 fs to 40 fs. For longer delays above 40 fs, the ionization yield is decreasing again.

**FIG. 6.**
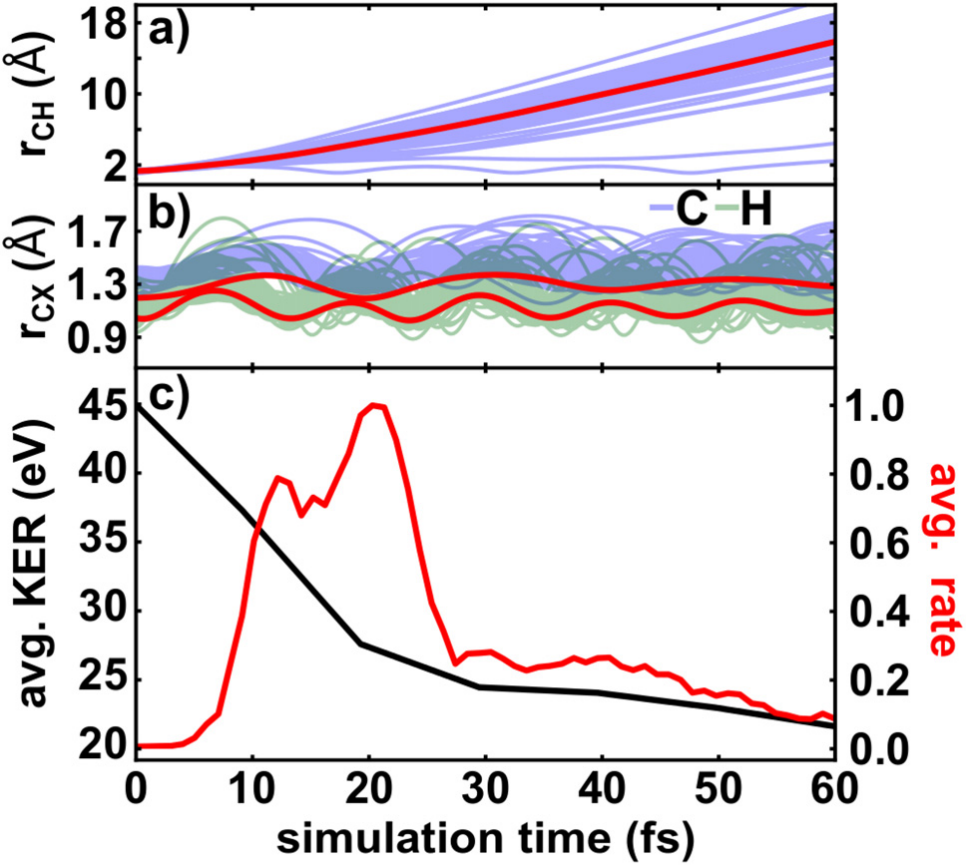
(a) The temporal evolution of the breaking CH bond in the first dissociative state of the acetylene dication shown in blue for all trajectories and in red for the averaged value. (b) The dynamics of the remaining CH bond (green) and the CC bond (blue) in the first dissociative state of the acetylene dication displayed for all trajectories and in red for the averaged values. (c) Calculated time-dependent KER and ionization rate of 20 exemplary trajectories propagating in first dissociative state of the acetylene dication.

For a better understanding of the observed enhanced ionization process, the nuclear dynamics in the first dissociative state of the acetylene dication (A^3^Π_u_) were simulated. The theoretical results are summarized in Fig. 6.

The temporal evolutions of all bond lengths in the dication of acetylene are displayed in Figs. 6(a) and 6(b). All 100 trajectories show deprotonation on the timescale of a few femtoseconds (<60 fs). On average, the leaving proton is around 2 Å away from the remaining C_2_H^+^ fragment after around 10 fs. In this fragment, an oscillation of the remaining bonds is observed. The CC bond has a period of around 20 fs and the CH bond of around 10 fs.

The averaged time-dependent KER and ionization rate of 20 exemplary trajectories are displayed in Fig. 6(c). The average KER is decreasing from about 45 eV to 22 eV within the first 60 fs. The ionization rate increases beginning from about 10 fs to 30 fs. For simulation times longer than 30 fs, the ionization yield is decreasing again. An enhanced ionization rate along the CH-bond occurs at the critical CH-bond length of around 2.0 Å after 10 fs, which corresponds well to the experimentally observed starting position of the yield enhancement. Compared to the simulations, the experimentally observed peak in EI is broader, which may be due to the used CASSCF method, which overestimates the steepness of the potential energy curve in the dication state leading to a faster nuclear motion. Similar to the simulations in the cation, another reason might be that the simulation includes only one single intermediate state. This is a simplification, since in reality several dissociative states with different potential energy surfaces may be populated. As the time to reach the critical bond distance can vary for each state, the peak becomes broader.

In order to get more insight into the EI process, also the three-fold coincidence channels C_2_H_2_^3+^ → H^+^ + C^+^ + CH^+^ and C_2_H_2_^3+^ → H^+^ + H^+^ + CC^+^ are investigated with respect to signs of enhanced ionization.

**FIG. 7.**
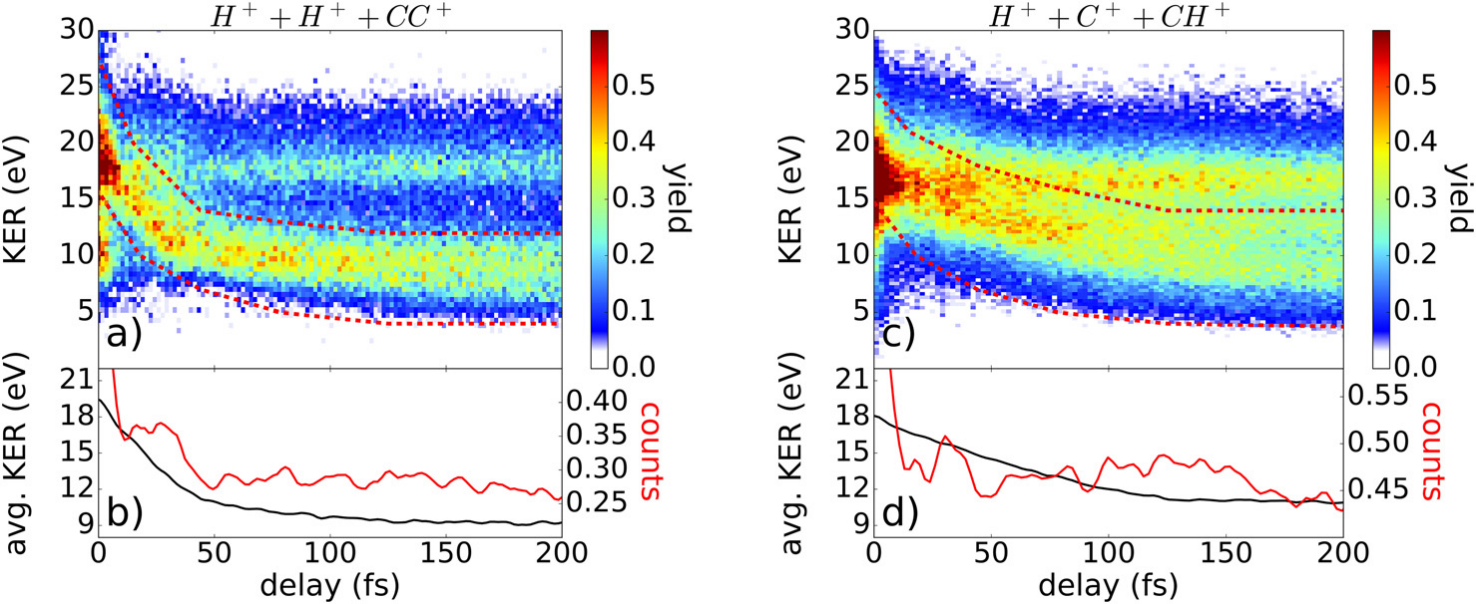
Ionization yield as a function of KER and delay of the coincidence channels C_2_H_2_^3+^ → H^+^ + H^+^ + CC^+^ and C_2_H_2_^3+^ → H^+^ + C^+^ + CH^+^ in (a) and (c), respectively. The one-dimensional plot in (b) and (d) represents the normalized KER-integrated signal including all counts within the dissociative area (red lines) and the average KER of the dissociative events [areas in between red dashed lines in (a) and (c)] as a function of the delay (black lines).

In Figs. 7(a) and 7(c), the ionization yield is depicted as a function of the delay for both three-fold coincidence channels. Similar to the four-fold coincidence channel, most of the molecules dissociate. These dissociating molecules are selected by the red marked filter and further analyzed in (b) and (d) with respect to their relative counts and average KER. In Fig. 7(b), a peak in the ionization yield is observed in between 10 and 40 fs. In comparison to the four-fold coincidence channel, here, the peak can be attributed to a pure CH-bond elongation. This suggests that also the EI peak seen in Fig. 5(b) originates from CH-bond stretching.

In Fig. 7(d), a small peak around 30 fs is observed, which fits well to the previous observations of enhanced ionization. Regarding the temporal evolution of average KER, it becomes obvious that the C_2_H_2_^3+^ → H^+^ + H^+^ + CC^+^ channel dissociates faster than the C_2_H_2_^3+^ → H^+^ + C^+^ + CH^+^ channel. Both observations can be explained by the fact that in the C_2_H_2_^3+^ → H^+^ + C^+^ + CH^+^ channel, not only the CH-bond but also the CC-bond is elongated. Thus, the temporal evolution of the dissociation process and therefore also the enhanced ionization is washed out and hence is not as well visible as in the C_2_H_2_^3+^ → H^+^ + H^+^ + CC^+^ channel.

## IV. SUMMARY

A nuclear wavepacket in the acetylene cation was temporally resolved via a pump-probe experiment. The pump pulse can either initiate a vibrational motion in the cation, which is observed in two different two-fold coincidence channels or it excites the molecule to a repulsive state leading to dissociation. The underlying processes were explained with the help of dynamic simulations and *ab-initio* calculations.

In the case of the bound cations, vibrational wave-packet motion could be imaged via the periodic modulation of the ionization rate. Periods of 25.5 ± 0.5 fs for the deprotonation channel and 26.7 ± 0.9 fs for the isomerization channel were extracted. The simulations of the ionization rate indicate that the observed modulation is dominantly determined by the symmetric CC-stretching normal mode in the ground state of the cation. Contributions from higher lying bound states in the cation are possible. We observe that the ionization rate is enhanced for elongated CC- and CH-bonds. For the dissociating acetylene dication, the process of enhanced ionization was measured experimentally via three- and four-fold coincidence channels to occur between 10 fs and 40 fs. In contrast to the bound cation state, the enhanced ionization can be clearly attributed to the deprotonation coordinate of acetylene. An enhanced ionization rate along the CH-bond occurs at the critical bond distance of 2.0 Å after 10 fs, which corresponds well to the experiment.

We have shown that reaction microscopy is a powerful tool for gaining detailed time-dependent information of enhanced ionization and related strong-field phenomena in complex molecules. Combining this information with the results from dynamic simulations and *ab-initio* calculations, the underlying ultrafast processes can be elucidated.

## Figures and Tables

**FIG. 8.**
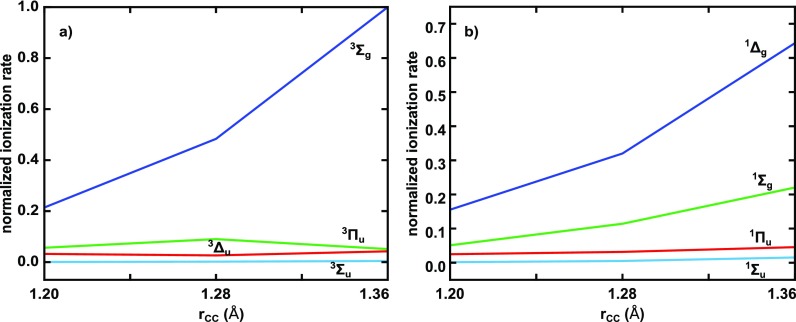
The normalized ionization rates along the CC stretching mode in the cationic ground state (X^2^Π_u_) calculated with the TD-RIS approach. The individual contributions of the first four triplet states (a) and of the first four singlet states (b) are shown.

**FIG. 9.**
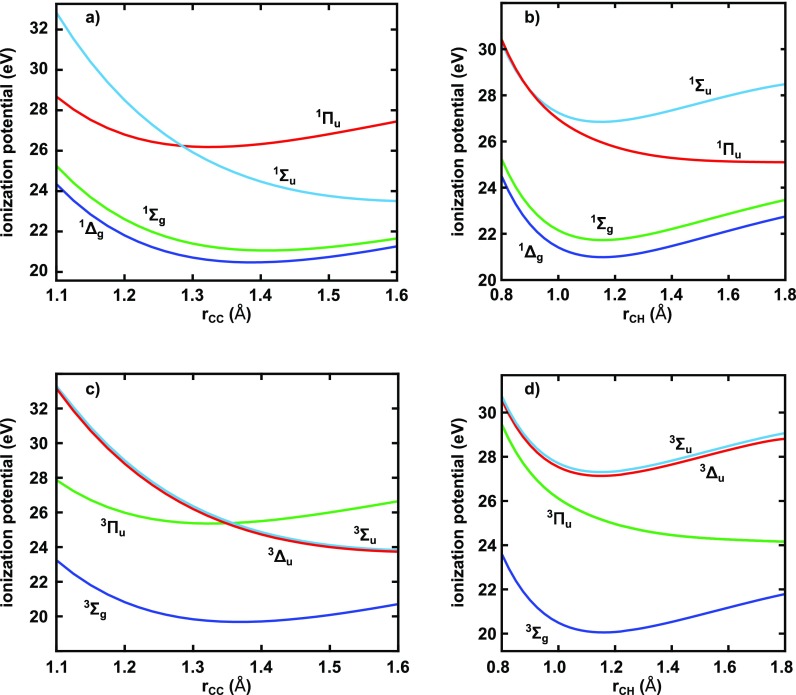
Potential energy curves along the CC [(a) and (b)] and the CH [(c) and (d)] bonds for the first four singlet [(a) and (b)] and for the first four triplet states [(c) and (d)] of the dication of acetylene. All energies are given relative to the ground state (X^2^Π_u_) energy of the cation.

**FIG. 10.**
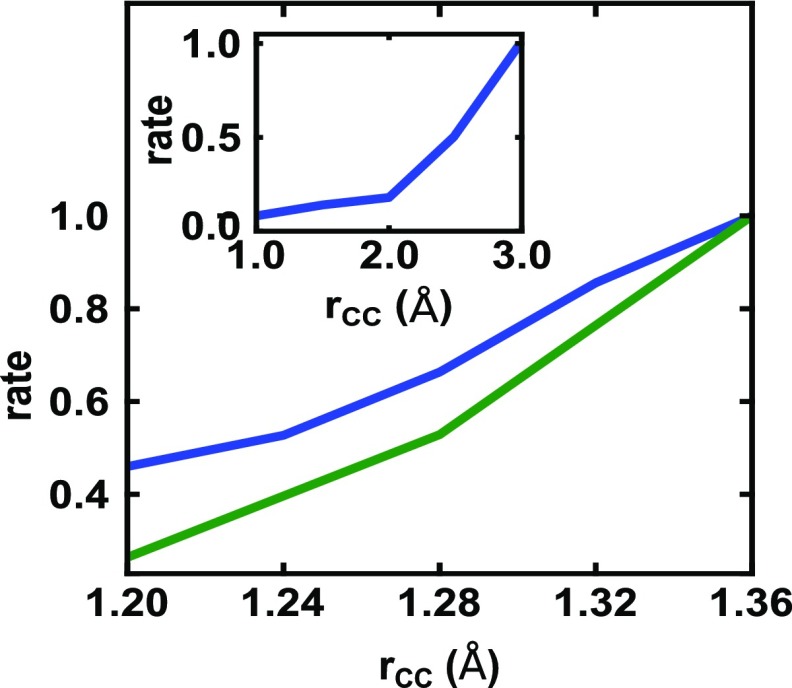
Normalized ionization rate along the CC stretching mode in the cationic ground state (X^2^Π_u_) for the current ansatz (blue) used and the TD-RIS approach (green). In the inset, the normalized ionization rate for the current ansatz (blue) is extended up to 3.0 Å.

**FIG. 11.**
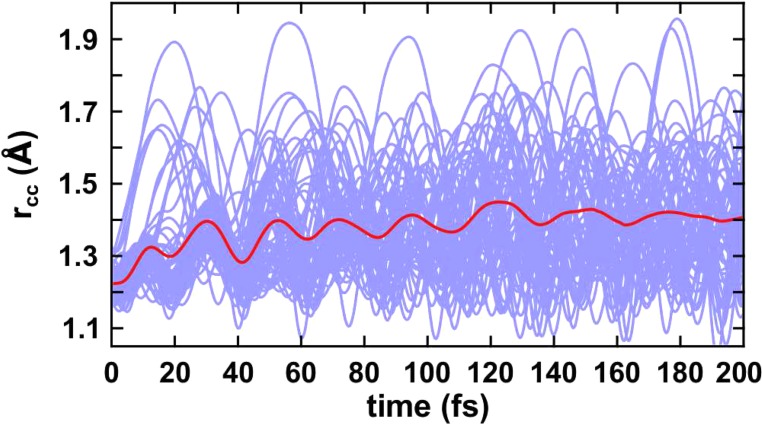
Simulated temporal dynamics of the CC bond in the A^2^Σ_g_^+^ state of the acetylene cation, shown in blue for all trajectories and in red for the averaged value.

## APPENDIX A: MR-CIS/TD-RIS CALCULATIONS

### 1. Calculated ionization rates

In the time-dependent resolution in ionic states (TD-RIS) approach, the ionization rates are calculated^43,44^ for ionization from acetylene cation to the dicationic singlet and triplet manifold, see Fig. 8. The electronic structure for the requisite elements of the simulation (i.e., transition densities, Dyson orbitals) was determined at the MR-CIS (multi-reference configuration interaction with single excitations) level of theory in combination with the augmented triple-zeta basis set (aug-cc-pVTZ), based on a full valence CASSCF reference wave function.^45,46^

### 2. Potential energy curves of acetylene dication

The potential energy curves for the first four singlet and the first four triplet states of the dication are shown in Fig. 9. The potential energy curves are calculated at the aug-cc-pVTZ/MR-CIS (multi-reference configuration interaction with single excitations) level of theory, based on a full valence CASSCF reference wave function.^45,46^

### 3. Comparison between on-the-fly simulations and TD-RIS

The ionization rate along the carbon-carbon (CC) stretching mode in the ground state of the cation (X^2^Π_u_) was calculated using both approaches, i.e.: (a) the non-adiabatic on-the-fly simulations and *ab-initio* calculations of ionization rates (see main text) and (b) TD-RIS. The first method takes into account all ionization pathways simultaneously, while the TD-RIS method distinguishes the pathways into individual states. For TD-RIS approach, only the range between 1.20 and 1.36 Å were calculated, whereas for current used ansatz CC distances from 1 up to 3 Å are evaluated. The normalized results are shown in Fig. 10; for comparison we summed over the four main TD-RIS contributions. Qualitatively, we find a good agreement; both methods show the same increase in the ionization rate with CC bond elongation. For CC distances larger than 2 Å, a significant increase in the ionization rate is observed (see inset).

## APPENDIX B: DYNAMIC SIMULATION IN THE A^2^Σ_g_^+^ STATE OF ACETYLENE

Compared to the experimental results, the simulated ionization rate for the ground state of the cation is in qualitatively good agreement. The vibrational period of around 23 fs is, however, slightly shorter than the experimental one with 25 fs. A reason might be that the simulation includes only one intermediate state, which is a simplification as likely not only a single state is populated but rather also a multitude of states. To estimate the possible influence of additionally populated states, dynamic simulations for the excited A^2^Σ_g_^+^ state were performed, see Fig. 11. In this state, the oscillation period of the CC stretching motion changes due to the isomerization process. Its period varies between 22 and 25 fs within the first 100 fs. Contribution from this state might explain the slightly longer period observed in the experiment. Additionally, the CASSCF methodology overestimates the steepness of the potential energy curve leading to a faster oscillation.

## References

[1] M. Kübel , R. Siemering , C. Burger , N. G. Kling , H. Li , A. S. Alnaser , B. Bergues , S. Zherebtsov , A. M. Azzeer , I. Ben-Itzhak , R. Moshammer , R. de Vivie-Riedle , and M. F. Kling , Phys. Rev. Lett. 116, 193001 (2016). 10.1103/PhysRevLett.116.193001 27232019

[2] H. J. Wörner , J. B. Bertrand , D. V. Kartashov , P. B. Corkum , and D. M. Villeneuve , Nature 466, 604 (2010). 10.1038/nature09185 20671706

[3] A. Assion , T. Baumert , M. Bergt , T. Brixner , B. Kiefer , V. Seyfried , M. Strehle , and G. Gerber , Science 282, 919 (1998). 10.1126/science.282.5390.919 9794756

[4] J. Wu , M. Meckel , L. P. H. Schmidt , M. Kunitski , S. Voss , H. Sann , H. Kim , T. Jahnke , A. Czasch , and R. Dörner , Nat. Commun. 3, 1113 (2012). 10.1038/ncomms2130 23047671

[5] T. Seideman , M. Y. Ivanov , and P. B. Corkum , Phys. Rev. Lett. 75, 2819 (1995). 10.1103/PhysRevLett.75.2819 10059413

[6] T. Zuo and A. D. Bandrauk , Phys. Rev. A 52, 2511 (1995). 10.1103/PhysRevA.52.R2511 9912637

[7] T. Ergler , A. Rudenko , B. Feuerstein , K. Zrost , C. D. Schröter , R. Moshammer , and J. Ullrich , Phys. Rev. Lett. 95, 93001 (2005). 10.1103/PhysRevLett.95.093001 16197211

[8] I. Ben-Itzhak , P. Q. Wang , A. M. Sayler , K. D. Carnes , M. Leonard , B. D. Esry , A. S. Alnaser , B. Ulrich , X. M. Tong , I. V. Litvinyuk , C. M. Maharjan , P. Ranitovic , T. Osipov , S. Ghimire , Z. Chang , and C. L. Cocke , Phys. Rev. A 78, 063419 (2008). 10.1103/PhysRevA.78.063419

[9] H. Xu , F. He , D. Kielpinski , R. T. Sang , and I. V. Litvinyuk , Sci. Rep. 5, 13527 (2015). 10.1038/srep13527 26314372 PMC4551962

[10] A. S. Alnaser , I. Litvinyuk , T. Osipov , B. Ulrich , A. Landers , E. Wells , C. M. Maharjan , P. Ranitovic , I. Bochareva , D. Ray , and C. L. Cocke , J. Phys. B: At., Mol. Opt. Phys. 39, 485 (2006). 10.1088/0953-4075/39/13/S21

[11] I. A. Bocharova , A. S. Alnaser , U. Thumm , T. Niederhausen , D. Ray , C. L. Cocke , and I. V. Litvinyuk , Phys. Rev. A 83, 013417 (2011). 10.1103/PhysRevA.83.013417

[12] I. Bocharova , R. Karimi , E. F. Penka , J. P. Brichta , P. Lassonde , X. Fu , J. C. Kieffer , A. D. Bandrauk , I. Litvinyuk , J. Sanderson , and F. Légaré , Phys. Rev. Lett. 107, 063201 (2011). 10.1103/PhysRevLett.107.063201 21902320

[13] X. Xie , S. Roither , M. Schöffler , H. Xu , S. Bubin , E. Lötstedt , S. Erattuphuza , A. Iwasaki , D. Kartashov , K. Varga , G. G. Paulus , A. Baltuška , K. Yamanouchi , and M. Kitzler , Phys. Rev. A 89, 023429 (2014). 10.1103/PhysRevA.89.023429

[14] C. Ellert and P. B. Corkum , Phys. Rev. A 59, R3170 (1999). 10.1103/PhysRevA.59.R3170

[15] N. Takemoto and A. Becker , Phys. Rev. A 84, 023401 (2011). 10.1103/PhysRevA.84.023401

[16] E. Lötstedt , T. Kato , and K. Yamanouchi , Phys. Rev. A 85, 041402 (2012). 10.1103/PhysRevA.85.041402

[17] E. Lötstedt , T. Kato , and K. Yamanouchi , J. Chem. Phys. 138, 104304 (2013). 10.1063/1.4794130 23514486

[18] S. Roither , X. Xie , D. Kartashov , L. Zhang , M. Schöffler , H. Xu , A. Iwasaki , T. Okino , K. Yamanouchi , A. Baltuška , and M. Kitzler , Phys. Rev. Lett. 106, 163001 (2011). 10.1103/PhysRevLett.106.163001 21599363

[19] X. Gong , Q. Song , Q. Ji , H. Pan , J. Ding , J. Wu , and H. Zeng , Phys. Rev. Lett. 112, 243001 (2014). 10.1103/PhysRevLett.112.243001 24996086

[20] S. Erattupuzha , C. L. Covington , A. Russakoff , E. Lötstedt , S. Larimian , V. Hanus , S. Bubin , M. Koch , S. Gräfe , A. Baltuška , X. Xie , K. Yamanouchi , K. Varga , and M. Kitzler , J. Phys. B: At., Mol. Opt. Phys. 50, 125601 (2017). 10.1088/1361-6455/aa7098

[21] I. Fischer , M. J. J. Vrakking , D. M. Villeneuve , and A. Stolow , Chem. Phys. 207, 331 (1996). 10.1016/0301-0104(95)00404-1

[22] T. Ergler , A. Rudenko , B. Feuerstein , K. Zrost , C. D. Schröter , R. Moshammer , and J. Ullrich , Phys. Rev. Lett. 97, 193001 (2006). 10.1103/PhysRevLett.97.193001 17155620

[23] S. De , I. A. Bocharova , M. Magrakvelidze , D. Ray , W. Cao , B. Bergues , U. Thumm , M. F. Kling , I. V. Litvinyuk , and C. L. Cocke , Phys. Rev. A 82, 13408 (2010). 10.1103/PhysRevA.82.013408

[24] S. Lochbrunner , W. Fuß , W. E. Schmid , and K.-L. Kompa , J. Phys. Chem. A 102, 9334 (1998). 10.1021/jp9809179

[25] K. Kosma , S. A. Trushin , W. Fuß , and W. E. Schmid , J. Phys. Chem. A 112, 7514 (2008). 10.1021/jp803548c 18661929

[26] W. Fuß , W. E. Schmid , and S. A. Trushin , J. Chem. Phys. 112, 8347 (2000). 10.1063/1.481478

[27] S. A. Trushin , K. Kosma , W. Fuß , and W. E. Schmid , Chem. Phys. 347, 309 (2008). 10.1016/j.chemphys.2007.09.057

[28] C. Burger , N. G. Kling , R. Siemering , A. S. Alnaser , B. Bergues , A. M. Azzeer , R. Moshammer , R. de Vivie-Riedle , M. Kübel , and M. Kling , Faraday Discuss. 194, 495 (2016). 10.1039/C6FD00082G 27711784

[29] M. Schultze , A. Wirth , I. Grguras , M. Uiberacker , T. Uphues , A. J. Verhoef , J. Gagnon , M. Hofstetter , U. Kleineberg , E. Goulielmakis , and F. Krausz , J. Electron Spectrosc. Relat. Phenom. 184, 68 (2011). 10.1016/j.elspec.2011.01.003

[30] B. Roos , P. R. Taylor , and P. E. M. Siegbahn , Chem. Phys. 48, 157 (1980). 10.1016/0301-0104(80)80045-0

[31] M. Kübel , C. Burger , R. Siemering , N. G. Kling , B. Bergues , A. S. Alnaser , I. Ben-Itzhak , R. Moshammer , R. de Vivie-Riedle , and M. F. Kling , Mol. Phys. 115, 1835 (2017). 10.1080/00268976.2017.1288935 27232019

[32] H. Li , N. G. Kling , T. Gaumnitz , C. Burger , R. Siemering , J. Schötz , Q. Liu , L. Ban , Y. Pertot , J. Wu , A. M. Azzeer , R. de Vivie-Riedle , H. J. Wörner , and M. F. Kling , Opt. Express 25, 14192 (2017). 10.1364/OE.25.014192 28789005

[33] H.-J. Werner , P. J. Knowles , G. Knizia , F. R. Manby , and M. Schütz , WIREs Comput. Mol. Sci. 2, 242 (2012). 10.1002/wcms.82

[34] H.-J. Werner , P. J. Knowles , G. Knizia , F. R. Manby , M. Schütz , P. Celani , W. Györffy , D. Kats , T. Korona , R. Lindh , A. Mitrushenkov , G. Rauhut , K. R. Shamasundar , T. B. Adler , R. D. Amos , A. Bernhardsson , A. Berning , D. L. Cooper , M. J. O. Deegan , A. J. Dobbyn , F. Eckert , E. Goll , C. Hampel , A. Hesselmann , G. Hetzer , T. Hrenar , G. Jansen , C. Köppl , Y. Liu , A. W. Lloyd , R. A. Mata , A. J. May , S. J. McNicholas , W. Meyer , M. E. Mura , A. Nicklass , D. P. O'Neill , P. Palmieri , D. Peng , K. Pflüger , R. Pitzer , M. Reiher , T. Shiozaki , H. Stoll , A. J. Stone , R. Tarroni , T. Thorsteinsson , and M. Wang , MOLPRO, version 2012.1 a package of ab initio programs, http://www.molpro.net (2012)

[35] W. J. Hehre , R. Ditchfield , and J. A. Pople , J. Chem. Phys. 56, 2257 (1972). 10.1063/1.1677527

[36] P. C. Hariharan and J. A. Pople , Theor. Chim. Acta 28, 213 (1973). 10.1007/BF00533485

[37] M. M. Francl , W. J. Pietro , W. J. Hehre , S. J. Binkley , M. S. Gordon , D. J. DeFrees , and J. A. Pople , J. Chem. Phys. 77, 3654 (1982). 10.1063/1.444267

[38] M. Barbatti , M. Ruckenbauer , F. Plasser , J. Pittner , G. Granucci , M. Persico , and H. Lischka , WIREs Comput. Mol. Sci. 4, 26 (2014). 10.1002/wcms.1158

[39] M. Barbatti , G. Granucci , M. Ruckenbauer , F. Plasser , J. Pittner , M. Persico , and H. Lischka , NEWTONX: a package for Newtonian dynamics close to the crossing seam, version 1.4, http://www.newtonx.org (2012)

[40] B. P. Fingerhut , S. Oesterling , K. Haiser , K. Heil , A. Glas , W. J. Schreier , W. Zinth , T. Carell , and R. de Vivie-Riedle , J. Chem. Phys. 136, 204307 (2012). 10.1063/1.4720090 22667560

[41] P. von den Hoff , I. Znakovskaya , S. Zherebtsov , M. F. Kling , and R. de Vivie-Riedle , Appl. Phys. B 98, 659 (2010). 10.1007/s00340-009-3860-x

[42] D. Ray , F. He , W. Cao , H. Mashiko , P. Ranitovic , K. P. Singh , I. Znakovskaya , U. Thumm , G. G. Paulus , M. F. Kling , I. V. Litvinyuk , and C. L. Cocke , Phys. Rev. Lett. 103, 223201 (2009). 10.1103/PhysRevLett.103.223201 20366092

[43] M. Spanner and S. Patchkovskii , Phys. Rev. A 80, 063411 (2009). 10.1103/PhysRevA.80.063411

[44] M. Spanner and S. Patchkovskii , Chem. Phys. 414, 10 (2013). 10.1016/j.chemphys.2011.12.016

[45] H. Lischka , R. Shepard , R. M. Pitzer , I. Shavitt , M. Dallos , T. Müller , P. G. Szalay , M. Seth , G. S. Kedziora , S. Yabushita , and Z. Zhang , Phys. Chem. Chem. Phys. 3, 664 (2001). 10.1039/b008063m

[46] H. Lischka , T. Müller , P. G. Szalay , I. Shavitt , R. M. Pitzer , and R. Shepard , WIREs Comput. Mol. Sci. 1, 191 (2011). 10.1002/wcms.25

[47] A. S. Alnaser , M. Kübel , R. Siemering , B. Bergues , N. G. Kling , K. J. Betsch , Y. Deng , J. Schmidt , Z. A. Alahmed , A. M. Azzeer , J. Ullrich , I. Ben-Itzhak , R. Moshammer , U. Kleineberg , F. Krausz , R. de Vivie-Riedle , and M. F. Kling , Nat. Commun. 5, 3800 (2014). 10.1038/ncomms4800 24806279

[48] H. Ibrahim , B. Wales , S. Beaulieu , B. E. Schmidt , N. Thiré , E. P. Fowe , É. Bisson , C. T. Hebeisen , V. Wanie , M. Giguére , J.-C. Kieffer , M. Spanner , A. D. Bandrauk , J. Sanderson , M. S. Schuurman , and F. Légaré , Nat. Commun. 5, 4422 (2014). 10.1038/ncomms5422 25034613

[49] Y. H. Jiang , A. Rudenko , O. Herrwerth , L. Foucar , M. Kurka , K. U. Kühnel , M. Lezius , M. F. Kling , J. Van Tilborg , A. Belkacem , K. Ueda , S. Düsterer , R. Treusch , C. D. Schröter , R. Moshammer , and J. Ullrich , Phys. Rev. Lett. 105, 263002 (2010). 10.1103/PhysRevLett.105.263002 21231652

[50] A. Rudenko , T. Ergler , B. Feuerstein , K. Zrost , C. D. Schröter , R. Moshammer , and J. Ullrich , Chem. Phys. 329, 193 (2006). 10.1016/j.chemphys.2006.06.038 17155620

[51] H. Niikura , D. M. Villeneuve , and P. B. Corkum , Phys. Rev. A 73, 21402 (2006). 10.1103/PhysRevA.73.021402

